# Supplementary material for: Epitaxial thin films of pyrochlore iridate Bi2+xIr2-yO7-δ: structure, defects and transport properties
Source: Sci Rep. 2017 Aug 10;7:7740. doi: 10.1038/s41598-017-06785-w (PMC5552750; doi:10.1038/s41598-017-06785-w)
Supplement: Supplementary file 1 — Supplementary Information [file 41598_2017_6785_MOESM1_ESM.pdf]

Supplementary Information

Epitaxial thin films of pyrochlore iridate $Bi_{2+x}Ir_{2-y}O_{7-\delta}$: structure, defects and transport properties

W. C. Yang[1,+], Y. T. Xie[2,+], W. K. Zhu[1,+], K. Park[2], A. P. Chen[3], Y. Losovyj[4], Z. Li[1,3], H. Liu[1], M. Starr[1], J. A. Acosta[1], C. G. Tao[2], N. Li[3], Q. X. Jia[3,5], J. J. Heremans[2], and S. X. Zhang[1,*]

[1]Department of Physics, Indiana University, Bloomington, Indiana 47405, USA
[2]Department of Physics, Virginia Tech, Blacksburg, Virginia 24061, USA
[3]Center for Integrated Nanotechnologies, Los Alamos National Laboratory, Los Alamos, 87545 USA
[4]Department of Chemistry, Indiana University, Bloomington, Indiana 47405, USA
[5]Department of Materials Design and Innovation, University at Buffalo, The State University of New York, Buffalo, NY 14260, USA
* sxzhang@indiana.edu
[+] these authors contributed equally to this work

1. X-ray diffraction (XRD) characterization of thin films

Figure S1 shows the XRD 2θ-ω patterns of the YSZ (111) substrate and some representative thin films. The (222), (333) and (444) peaks of the Bi-227 films are identified. In particular, the detection of (333) peak suggests good film quality. The Ir (111) peak is observed in the films grown at 10mTorr and 1mTorr, indicating the formation of iridium metal impurity at low P_{O2}. The film at 1mTorr is mainly composed of Ir metal, consistent with the EDX measurement (Figure S2(c)). The peaks denoted by '*' are due to Cu k_{β} and the other tiny sharp peaks are either due to the instrument or the substrate as they are observed in the bare substrate as well. Since the (222) peaks of some films are too close to the substrate (111) peak, preventing from an accurate determination of the diffraction angles, the d_{222} values in Figure 2(a) were calculated based on the (444) peaks, i.e. $d_{222} = 2d_{444}$.

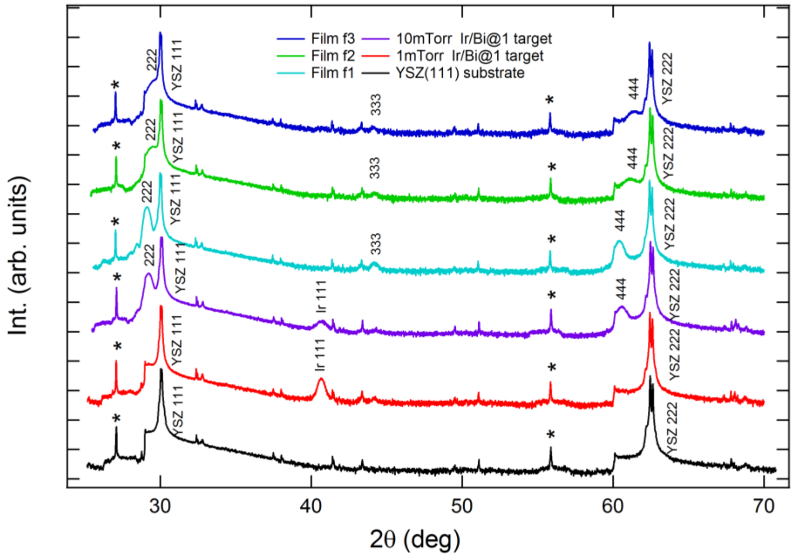

Figure S1. *XRD 2θ–ω scans of the YSZ substrate and the films grown at: P_{O2}= 50 mTorr, Ir/Bi@1 target (f1); P_{O2} = 50 mTorr, Ir/Bi@3 target (f2); P_{O2} = 15 mTorr, Ir/Bi@3 target (f3); P_{O2}= 10 mTorr, Ir/Bi@1 target; and P_{O2}= 1 mTorr, Ir/Bi@1 target. The vertical axis is in logarithmic scale and all patterns are corrected/calibrated against the substrate peak with a lattice constant of a_{ysz}=5.125 Å to determine the d-spacings of thin films.*

2. Energy-dispersive X-ray spectroscopy study of thin films

Energy-dispersive X-ray spectroscopy (EDX) was employed to characterize the chemical composition of thin films. Figure S2(a) shows a scanning electron microscope (SEM) image of a representative thin film (10 mTorr with an Ir/Bi@3 target). Figure S2(b) is the EDX spectrum which confirms the existence of Ir and Bi in the film. The Y, Zr and Hf signals are from the YSZ substrate which often contains Hf impurity. The atomic ratio of Ir/Bi is calculated in the AZtech software. Figure S2(c) is the EDX spectrum of a film grown at P_{O2} = 1 mTorr which has a negligible Bi signal.

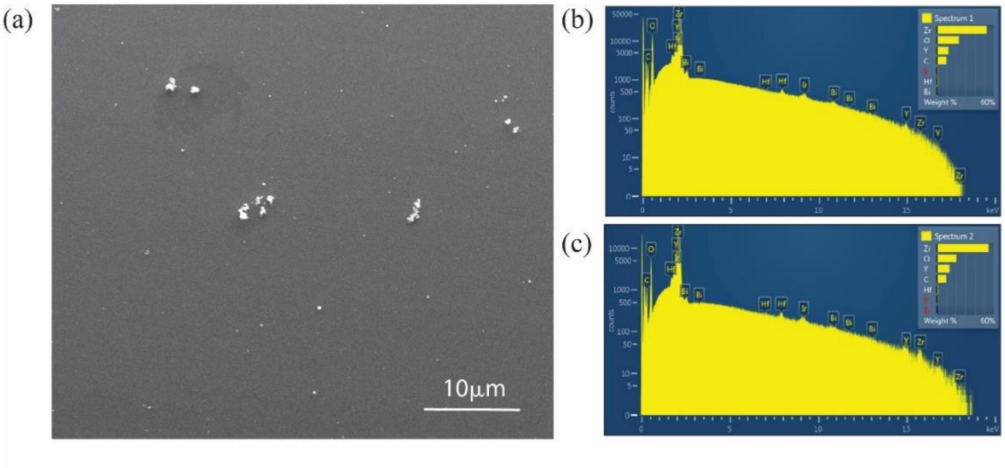

Figure S2. *(a) An SEM image and (b) EDX spectrum of a thin film grown at P_{O2} =10 mTorr using an Ir/Bi@3 target. (c) EDX spectrum of a film grown at P_{O2} = 1 mTorr using an Ir/Bi@1 target.*

3. X-ray photoelectron spectroscopy (XPS) characterization of film f2 ($\boldsymbol{P_{O2}}$ = 50 mTorr, Ir/Bi@3 target)

Figure S3 (a) and (b) show the XPS Ir and Bi spectra taken on film f2, respectively. The spectra were fitted using the procedure described in section 5.

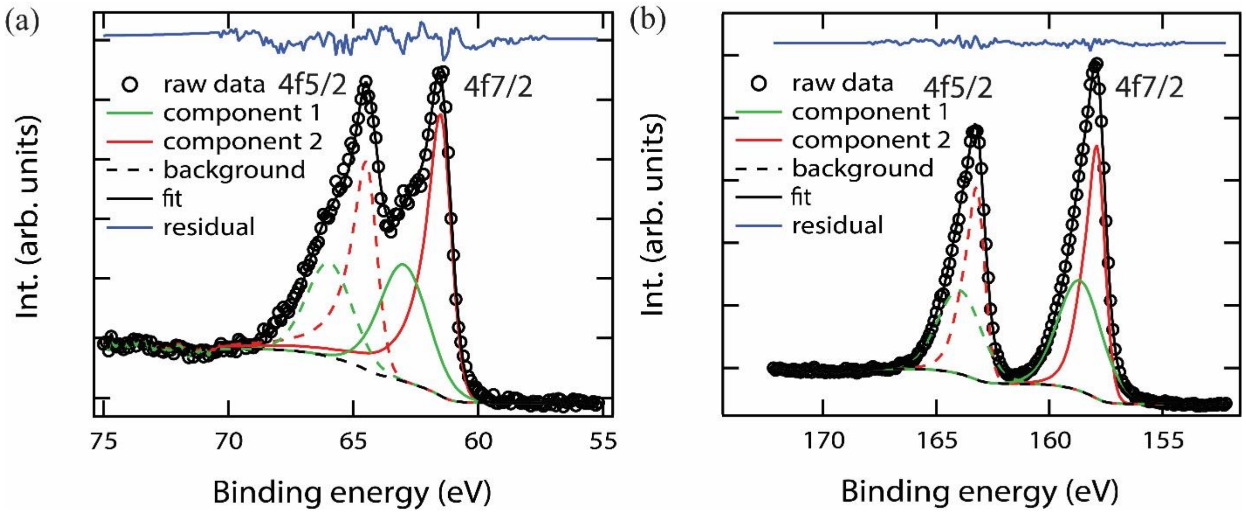

Figure S3. *XPS (a) Ir $4f_{5/2}$ and $4f_{7/2}$ spectra and (b) Bi $4f_{5/2}$ and $4f_{7/2}$ spectra of film f2. The spectra are fitted using a Gaussian lineshape GL(m) and a convolution of Gelius profile A(a,b,n) and GL(m).*

4. Comparison of XPS spectra between films and bulk IrO_2, Bi_2O_3 powder

XPS measurements were taken on standard IrO_2 and Bi_2O_3 powder as references. The peaks from thin films are on the higher side of the spectra comparing to IrO_2 and Bi_2O_3, suggesting lower oxidation states in the thin film samples.

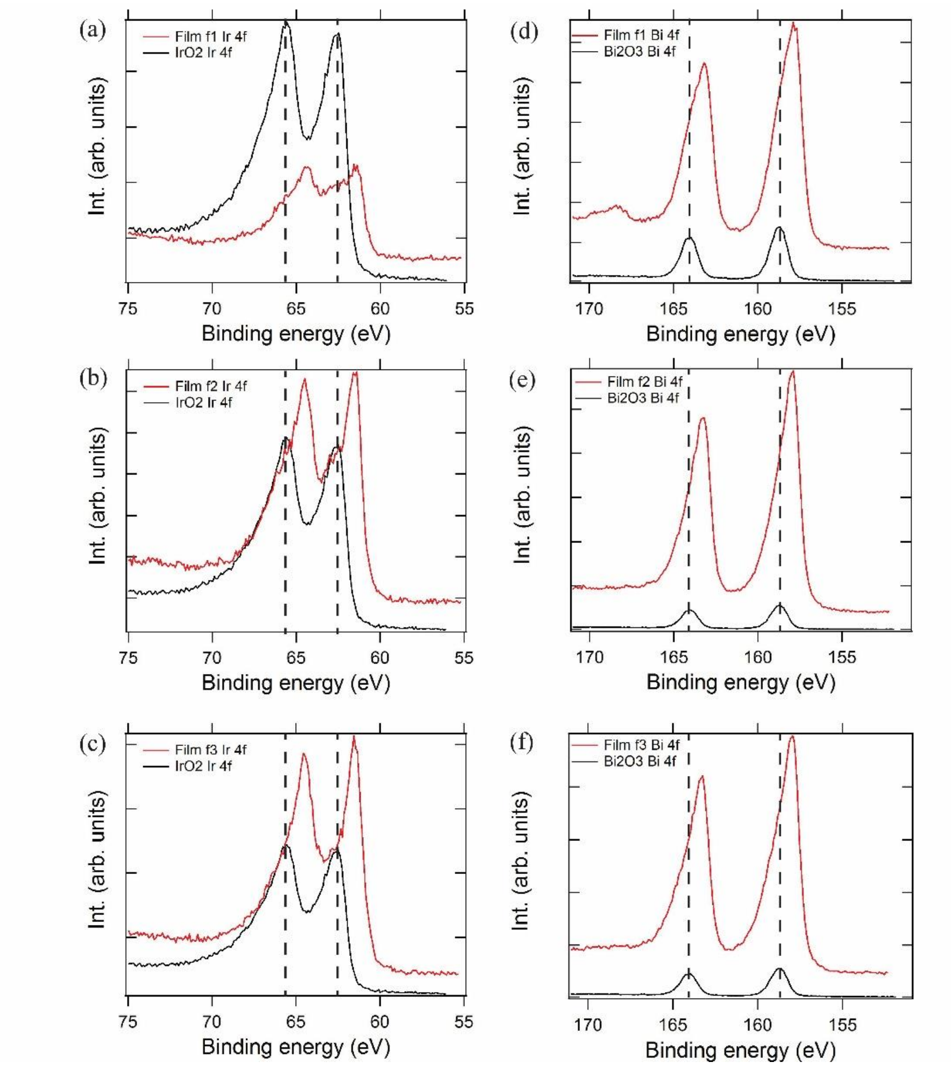

Figure S4. *Comparison of XPS spectra of IrO_2 and Bi_2O_3 with compound (a) IrO_2 and film f1 (b) IrO_2 and film f2 (c) IrO_2 and film f3 (d) Bi_2O_3 and film f1 (e) Bi_2O_3 and film f2 (f) Bi_2O_3 and film f3. The dashed lines indicate the binding energies of 4f5/2 and 4f7/2 states.*

5. Fitting of XPS spectra

The XPS spectra were fitted using CasaXPS software. The high binding energy peak was fitted using a symmetric Gaussian lineshape GL(m) while the lower one was fitted by an asymmetric lineshape. The asymmetric lineshape was described by the convolution of Gelius profile A(a,b,n) and Gaussian GL(m), in which the asymmetric part is characterized by A(a,b,n). In a typical fitting process, we choose one combination of parameters a and b in the Gelius profile and run the fitting from which we obtain the residual spectra, i.e. the difference between the experimental data and the fitting spectra. By repeating the procedure with different combinations of a and b, a residual STD as a function of parameter a and b is obtained. The parameters a and b are manually tuned in order to yield a minimum value in the residual STD curve. Figure S5 gives the residual STD versus parameter b with different a for f1 Ir spectrum, the best fit parameters are obtained when the residual function reaches a minimum value, in this case a=0.4 and b=0.6.

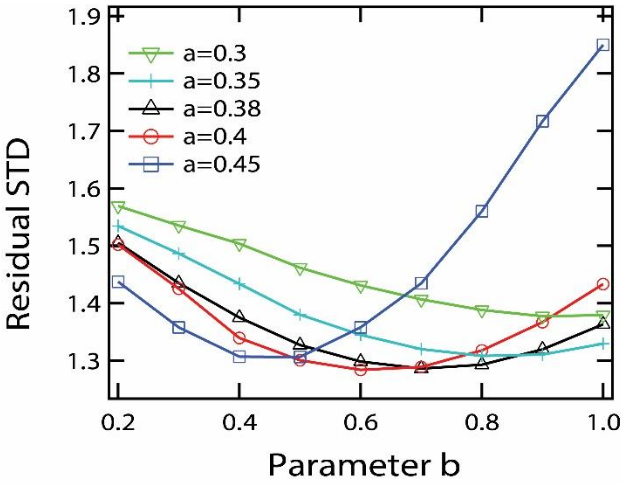

Figure S5. *Residual STD versus parameter b with different parameter a for the Ir spectrum of film f1.*

6. Unit cells of $Bi_2Ir_2O_7$ with and without point defect

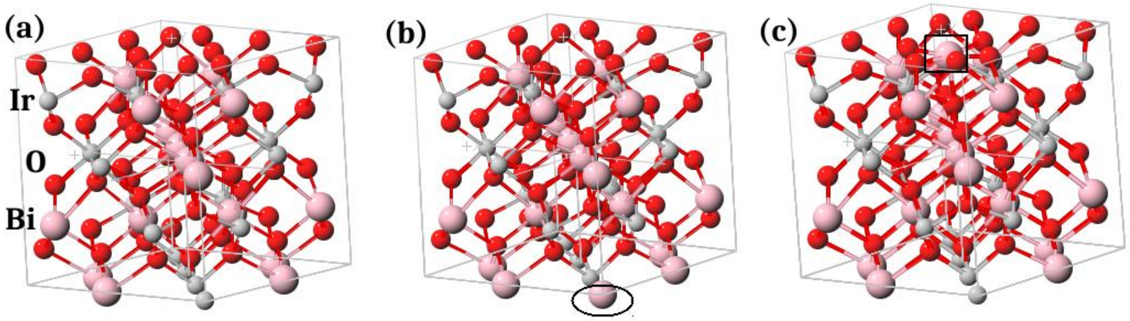

Figure S6. *The 88-atom unit cells of (a) a perfect crystal, (b) one* Bi_{Ir} *antisite (indicated by the circled Bi atom at bottom), and (c) one interstitial Bi atom among the O atoms (indicated by the boxed Bi atom on top). Although a unit cell of perfect crystal consists of 22 atoms, for clear comparison with (b) and (c), an 88-atom unit cell is shown for (a).*
